# On preserving anatomical detail in statistical shape analysis for clustering: focus on left atrial appendage morphology

**DOI:** 10.3389/fnetp.2024.1467180

**Published:** 2024-10-10

**Authors:** Matthew T. Lee, Vincenzo Martorana, Rafizul Islam Md, Raphael Sivera, Andrew C. Cook, Leon Menezes, Gaetano Burriesci, Ryo Torii, Giorgia M. Bosi

**Affiliations:** ^1^ UCL Mechanical Engineering, University College of London, London, United Kingdom; ^2^ Department of Economics Management, and Statistics, University of Palermo, Palermo, Italy; ^3^ UCL Medical Physics and Biomedical Engineering, University College of London, London, United Kingdom; ^4^ UCL Institute of Cardiovascular Science, University College of London, London, United Kingdom; ^5^ Biomedical Research Centre, National Institute for Health and Care Research, University College London Hospitals, London, United Kingdom; ^6^ Bioengineering Group, Ri.MED Foundation, Palermo, Italy

**Keywords:** statistical shape analysis, hierarchical clustering, left atrial appendage (LAA), atrial fibrillation, principal component analysis -PCA, clustering performance evaluation, segmentation (image processing)

## Abstract

**Introduction:**

Statistical shape analysis (SSA) with clustering is often used to objectively define and categorise anatomical shape variations. However, studies until now have often focused on simplified anatomical reconstructions, despite the complexity of studied anatomies. This work aims to provide insights on the anatomical detail preservation required for SSA of highly diverse and complex anatomies, with particular focus on the left atrial appendage (LAA). This anatomical region is clinically relevant as the location of almost all left atrial thrombi forming during atrial fibrillation (AF). Moreover, its highly patient-specific complex architecture makes its clinical classification especially subjective.

**Methods:**

Preliminary LAA meshes were automatically detected after robust image selection and wider left atrial segmentation. Following registration, four additional LAA mesh datasets were created as reductions of the preliminary dataset, with surface reconstruction based on reduced sample point densities. Utilising SSA model parameters determined to optimally represent the preliminary dataset, SSA model performance for the four simplified datasets was calculated. A representative simplified dataset was selected, and clustering analysis and performance were evaluated (compared to clinical labels) between the original trabeculated LAA anatomy and the representative simplification.

**Results:**

As expected, simplified anatomies have better SSA evaluation scores (compactness, specificity and generalisation), corresponding to simpler LAA shape representation. However, oversimplification of shapes may noticeably affect 3D model output due to differences in geometric correspondence. Furthermore, even minor simplification may affect LAA shape clustering, where the adjusted mutual information (AMI) score of the clustered trabeculated dataset was 0.67, in comparison to 0.12 for the simplified dataset.

**Discussion:**

This study suggests that greater anatomical preservation for complex and diverse LAA morphologies, currently neglected, may be more useful for shape categorisation via clustering analyses.

## 1 Introduction

**FIGURE 1:**
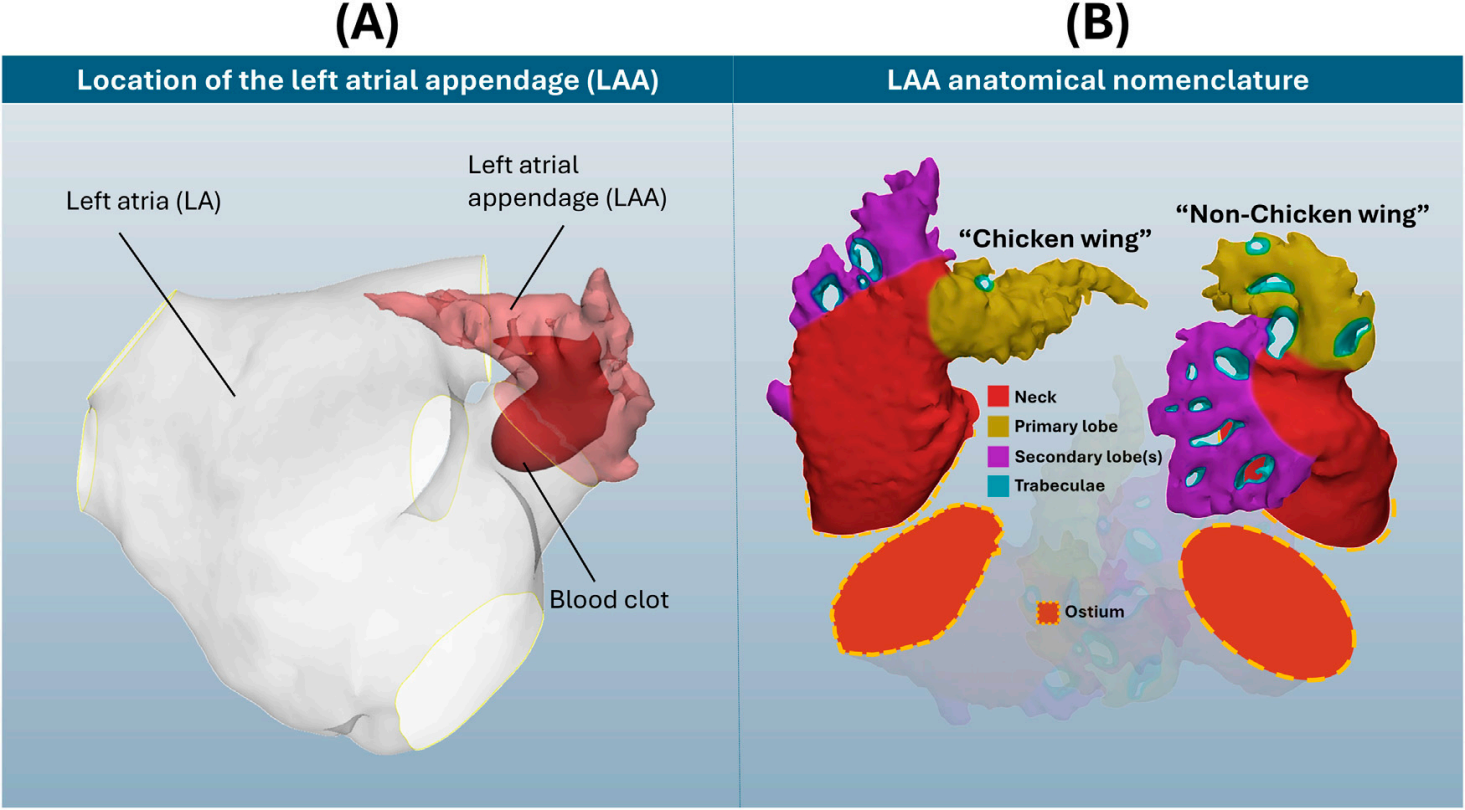
**(A)** Location of the LAA on the left atria, with blood clot representation. **(B)** Visual display of two selected LAA cases, with anatomical nomenclature of ostium, neck, primary and secondary lobes and trabeculae. Note how these example LAA anatomies differ considerably in both shape and detail, which does not include the full breadth of LAA morphological variation.

*Shape* is mathematically defined as “all the geometrical information that remains when location, scale and rotational effects are filtered out from an object” (Kendall, 1977). Shape analysis refers to a wide variety of mathematical/computational methods that may be used to identify the geometrical similarities and differences within a cohort of shapes. In recent years, there has been an adoption of statistical shape analysis (SSA) applications to human organs and vessels; this type of analysis is considered to be a step up from clinical morphometry due to greater objectivity and/or the identification and quantification of subtle geometrical information (Goparaju et al., 2022; Cerrolaza et al., 2019). Of the many such studied anatomies, the left atrial appendage (LAA), a natural closed-ended outgrowth of the left atrium (Figure 1A), stands out for its morphological complexity (in terms of both macro-shape and anatomical intricacy) and high diversity among different subjects.

The LAA is considered the origin of up to 91% of all left atrial thrombi during atrial fibrillation (AF) (Blackshear and Odell, 1996), the most common cardiac arrhythmia, affecting 59 million people worldwide and with increasing prevalence in older patients (about 20%–33% of risk above 45 years of age) (Linz et al., 2024). LAA shape category for thrombosis risk assessment is typically determined through clinical classification systems. The most used classification system defines 4 LAA types–chicken wing, windsock, cauliflower and cactus (Wang et al., 2010; Korhonen et al., 2015) (in debatable order of lower to greater thrombosis risk (Musotto et al., 2022; Bosi et al., 2018)) – that may be determined through morphometric measurements of LAA length, bending angle and number of lobes. However, this categorisation is commonly subject to clinical disagreement, with a study revealing consensus among three expert clinicians to be only reached in 28.9% of cases (Wu et al., 2019). Instead, as labelled in Figure 1B, more recent clinical (Yaghi et al., 2020) and SSA (Juhl et al., 2024; Ahmad et al., 2024) studies suggest that LAA categorisation may be primarily approached as chicken wing-like (characterized by high length and bending angle), and non-chicken wing-like.

Conventional LAA anatomical nomenclature (Barbero and Ho, 2017) is also displayed in Figure 1B for these two shapes: divided into ostium, neck, primary and secondary lobes, and trabeculae. The ostium refers to the entry-point for blood flow, dividing the left atrium from the LAA. The neck refers to the main body volume above the ostium, which connects to both the primary lobe and tip, as well as secondary lobes along the LAA length. Trabeculae, appearing as holes that pass fully through the LAA blood pool, are devoid of blood flow due to pectinate muscle fibres connecting opposing walls of the appendage chamber. As seen in Figure 1B, LAA anatomies may differ considerably in both their macro-shape and intricate anatomical detail, i.e., trabeculae.

The inclusion of intricate anatomical details, such as LAA trabeculae, may further improve thrombosis risk assessment of LAA shape. In a normally functioning human heart, blood passes through the complex anatomy of the LAA in atrial diastole and washes out thoroughly during atrial systole. In AF conditions, the presence of these fine LAA morphological features has a much greater impact on the fluid mechanics–with greater thrombosis risk around trabeculae and towards the tips of lobes (Musotto et al., 2022). Furthermore, a recent computational study of LAA morphological parameters (Martorana et al.) suggests that the quantification of trabeculae may also be useful for shape analysis.

To better evaluate LAA shape than current clinical classification systems, studies have suggested various approaches towards in-depth LAA morphological understandings. Multivariate morphometric LAA shape analyses, to which haemodynamic measurements may also be combined (Pons et al., 2022), are useful to represent thrombosis risk with respect to simple shape measurements. More in-depth approaches, i.e., LAA SSA, have the additional advantage of preserving LAA anatomical variation in 3D mesh formats and outputting novel LAA categorical shapes (Goparaju et al., 2022; Juhl et al., 2024; Ahmad et al., 2024). SSA is based on the geometric correspondence of entire *shapes* (Kendall, 1977), where similarly shaped objects have greater correspondence (and *vice versa*), that is defined by the particular SSA implementation. LAA SSA representation for categorisation has been defined both explicitly with point correspondence (Goparaju et al., 2022; Juhl et al., 2024) and with implicit techniques (Goparaju et al., 2022; Ahmad et al., 2024). Building upon these SSA frameworks, such studies may then propose a computational categorisation of their LAA shape representations. This categorisation may be defined by hard (Ahmad et al., 2024; Goparaju et al., 2018) and soft (Juhl et al., 2024; Slipsager et al., 2019) clustering approaches, as well as non-clustering dimensionality reduction (Goparaju et al., 2022).

Despite multiple advances in LAA SSA (Goparaju et al., 2022; Juhl et al., 2024; Ahmad et al., 2024; Goparaju et al., 2018; Slipsager et al., 2019; Bhalodia et al., 2010; Bieging et al., 2021; Adams et al., 2023; Adams et al., 2022; Cates et al., 2015), no study has yet investigated the impact of intricate LAA morphological features such as trabeculae, surface roughness and tertiary lobe structure on LAA shape category definition. As key morphological components for the assessment of thrombosis risk, this study proposes that these features may also provide morphological information suitable for LAA shape categorisation (focussing on LAA SSA for clustering analysis). Therefore, this study compares LAA shape categorisation determined via hard clustering of LAA SSA models from fully trabeculated versus simplified datasets, suggesting that intricate anatomical detail (that includes trabeculations) provides additional analytical value for clustering LAA shape. This study does not aim to develop a new LAA classification scheme, but rather focus on the importance of preserving these anatomical details for clustering purposes.

## 2 Materials and methods

### 2.1 Image and mesh processing

**FIGURE 2:**
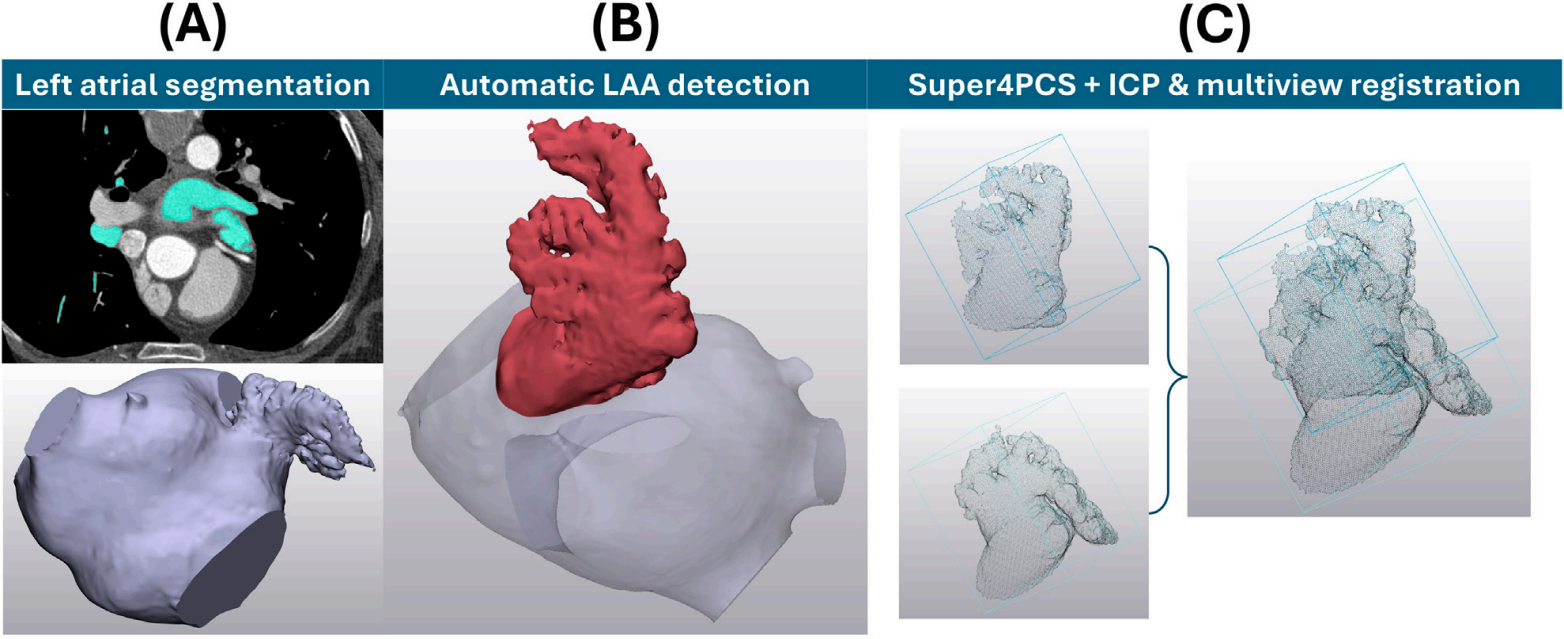
**(A)** LAA mesh acquisition and processing prior to SSA and clustering. The upper far left shows an example slice of the CT image stack to achieve the lower left atrial segmentation. **(B)** The LAA position determined through a fully automatic detection algorithm (Martorana et al.). **(C)** Two examples of LAA point clouds before and after alignment through Super4PCS registration, followed by ICP & multiview registration of all possible pairs.

85 clinical computerised tomography (CT) scans were used with informed consent by University College London Hospital (UCLH), consisting of non-AF patients examined for moderate coronary disease. The average participant age was 61.5 years, with 48 of the 85 of male sex. As this dataset is composed of control cases, not associated with thromboembolic risk, this study focuses on anatomical detail. Images are 512 × 512 pixels, with a pixel spacing of 0.488 mm × 0.488 mm, and a slice thickness of 0.625 mm acquired with the GE Discovery STE scanner. The manual segmentation protocol of full left atria was adapted from previous studies (Bosi et al., 2018; Capelli et al., 2012) to include measurements of contrast-to-noise ratio (CNR) and signal-to-noise ratio (SNR), following clinically recommended protocols (Marques et al., 2018), to ensure image (and hence later LAA shape) viability (Figure 2A). To summarise this process briefly, following calculation of CNR and SNR, 85 segmentation masks were generated in Mimics 24.0 (Materialise, Belgium) from the dye contrast threshold. These masks were manually processed by a segmentation expert to select only left atrial structures, including the LAA, pulmonary vein trunks and a mitral plane. After segmentation, each of the 85 left atria was evaluated by an expert cardiac anatomist to focus on chicken wing and non-chicken wing labels only. 21 LAAs were categorised as chicken wing and the remaining 64 as non-chicken wing.

Then, the full left atria, as surface models, were meshed using triangular elements of 0.5 mm edge length for subsequent LAA definition. To keep the process as objective as possible and preserve all anatomical details, the following approaches were taken. To ensure an objective definition of LAA ostial planes (conventionally defined through subjective manual assessment (Hołda et al., 2017)), a fully automatic LAA detection algorithm (Martorana et al.) was applied to all 85 segmented anatomies (Figure 2B). Briefly, this LAA detection method is based of distance analysis of computationally skeletonised left atria to automatically identify the LAA ostial plane, thus allowing LAA detection (Martorana et al.). To ensure normalisation across all detected LAAs, each mesh was then scaled to the same arbitrary volume (6,000 mm^3^, close to the average mesh volume). Global registration of the detected LAAs was performed via the Super4PCS algorithm (Mellado et al., 2014) to a single case, followed by local iterative closest point (ICP) (Rusinkiewicz and Levoy, 2001) and multiview registration (Pulli, 1999) across the full dataset (Figure 2C). Local ICP and multiview registration were repeated until all possible pairs fell within alignment distance. For 2000 sample points describing each anatomy chosen at each ICP iteration, the chosen minimal starting distance was 10 mm, reduced iteratively so that 80% of the samples would lie at a distance lower than 0.5 mm. Up to this point, no LAA structural definition was lost (i.e., shapes are fully inclusive of objectively defined LAA ostia, full surface structure, bending and anatomical lobes and trabeculae), ensuring that LAA shapes match ‘all the geometrical information that remains when location, scale and rotational effects are filtered out from an object, as per Kendall’s definition of shape (Kendall, 1977).

### 2.2 Simplified dataset generation

**FIGURE 3:**
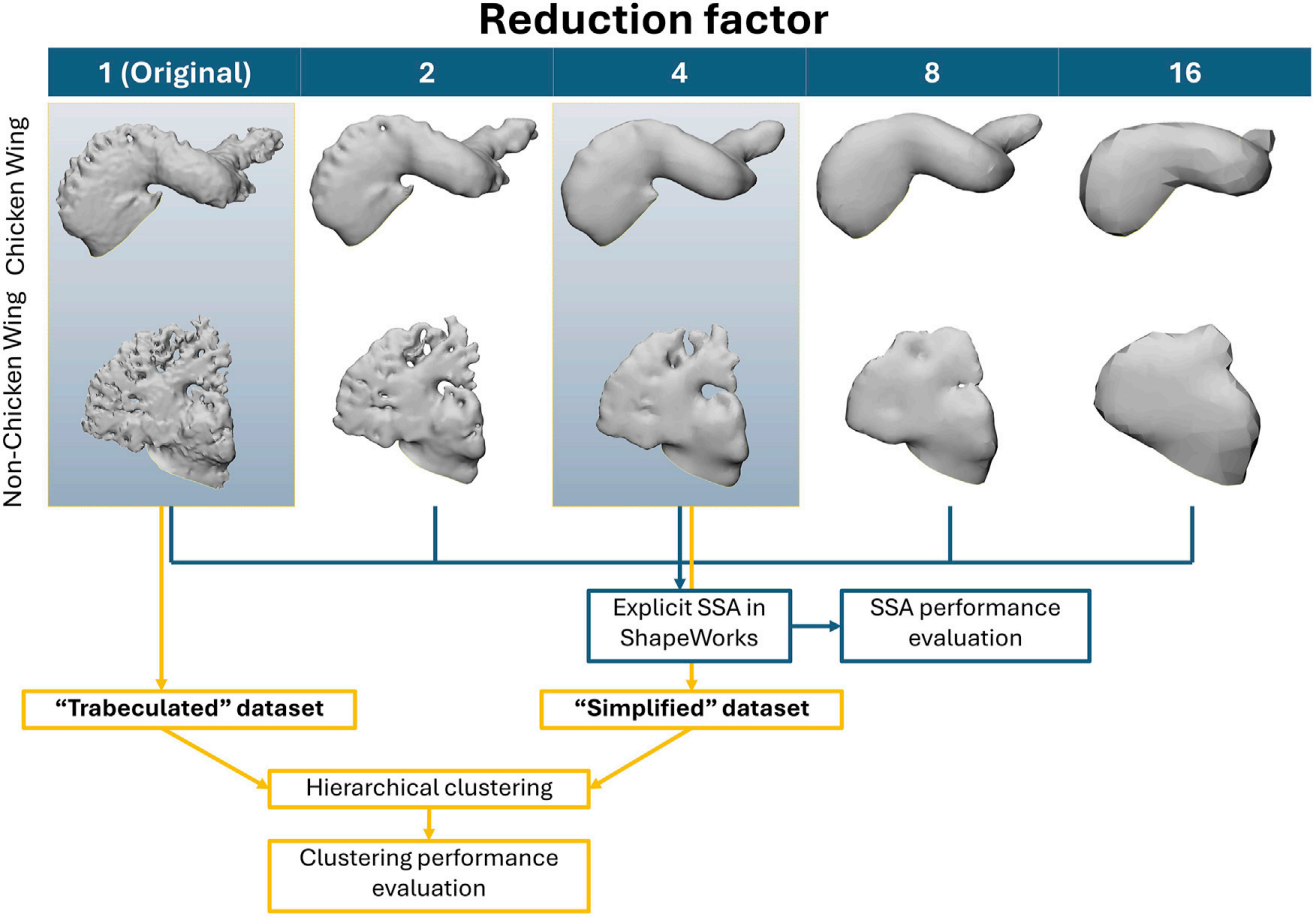
The simplified meshes (left to right) for two examples of LAA chicken wing and non-chicken wing morphologies from original trabeculated reconstruction, until full sample reduction. Note the visual loss in LAA trabeculae by 4-times sample reduction, and visual lobar definition loss by 8-times sample reduction. The data flow for the subsequent SSA and clustering methodology is also displayed in the bottom half of the figure–with SSA of all five datasets to determine SSA performance with greater sample reduction, followed by clustering comparison between the trabeculated dataset and one simplified dataset (4-times sample reduction).

Based on the surface mesh generated for the 85 LAAs, simplified datasets of the registered LAA meshes were generated in MeshLab (Cignoni et al., 2008). Poisson surface reconstruction creates watertight surfaces from point sets with oriented surface normals, with set reconstruction depths corresponding to effective voxel resolutions (Kazhdan and Hoppe, 2013). To simplify the intricate meshes, a reduction factor of 2-times, 4-times, 8-times and 16-times was first applied to the point sets of LAA meshes in the original trabeculated dataset, with preservation of the original surface normals. To sequentially reduce intricate features such as trabeculae for surface reconstruction, the minimum sampling density was set as the reduction factor for each simplified dataset. To ensure less reconstruction bias due to the reduced number of points, the surface reconstruction depth *d* (which corresponds to solving on a voxel grid whose resolution is no larger than (2^*d*^)^3^ (Kazhdan and Hoppe, 2013)) was specified for each simplification as equal to 8, 7, 6 and 5. The simplified variations of the intricate dataset are shown in Figure 3: LAA surface reconstruction with 4-times reduction results in fully removed trabeculae; further reductions may lead to greater loss in lobar definition.

### 2.3 Statistical shape analysis

**FIGURE 4:**
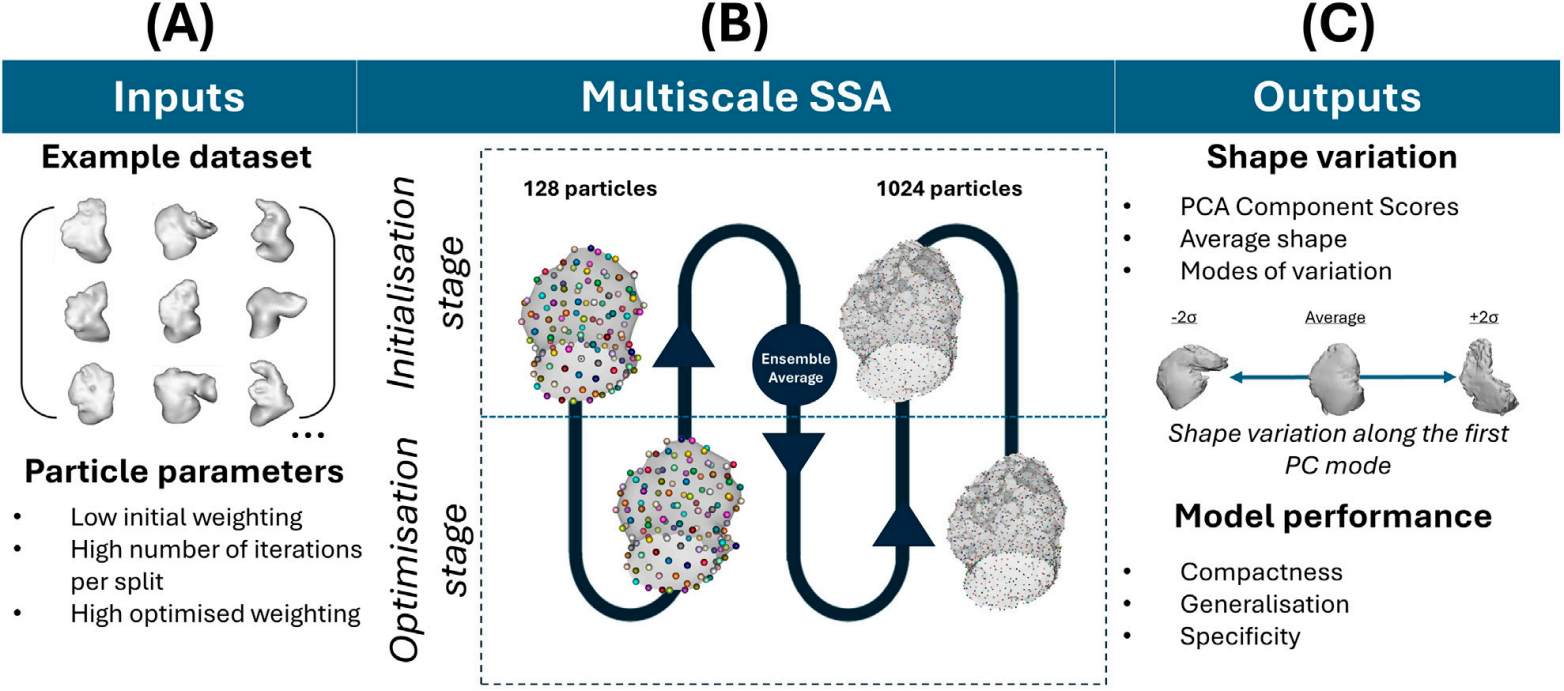
SSA workflow in ShapeWorks. **(A)** Refers to the input dataset and applied SSA parameters. **(B)** Refers to the SSA process, which is multiscale in the initialisation and optimisation of particle placements, with increasing particles’ number **(C)** Refers to the outputs of the SSA (i.e., the PCA component scores after particle optimisation, the average shape and its variations) and the model performance evaluation metrics.

LAA SSA was applied with the explicit method in ShapeWorks software, the most commonly studied “off-the-shelf” software for LAA shape analysis (Goparaju et al., 2022; Goparaju et al., 2018; Bhalodia et al., 2010; Bieging et al., 2021; Adams et al., 2023; Adams et al., 2022). All analyses were run on an AMD Ryzen 9 7950X3D 16-Core Processor, 4201 Mhz, 16 Core(s), 32 Logical Processor(s). The workflow for the SSA is laid out in Figure 4 and described below. The SSA model was run with 1,024 particles in multiscale from 128 (so that the initialisation and optimisation of particle position is rerun for each particle split), and principal component analysis (PCA) of the final particle correspondences was computed. Parameter selection (featuring a low initial weighting of particle position with a very high iteration number per particle split, and a high final optimised weighting (Cates et al., 2017)) was iteratively adjusted to balance SSA model evaluation metrics of compactness, generalisation and specificity (Davies, 2002) as implemented by ShapeWorks (Shape Model Evaluation). Briefly, compactness score $C\left(n_{m}\right),$ the degree to which a model has captured the morphological variation within a dataset, is defined as the sum of the eigenvalues $\lambda_{i}$ up to the selected number of PC modes $n_{m}$ , summarised as: $C\left(n_{m}\right)=\sum_{i=1}^{n_{m}}\lambda_{i}$ . Generalisation score $\hat{G}\left(n_{m}\right)$ , a measure of a SSA model’s ability to represent unseen shapes from a given dataset, may be quantified with the approximation error (Euclidean distance, in mm) between any held-out shape instance $x_{j}$ and its corresponding SSA model reconstruction ${\tilde{x}}_{j}$ , summarised as $\hat{G}\left(n_{m}\right)=\frac{1}{n_{s}}\sum_{j=1}^{n_{s}}\left\|x_{j}-{\tilde{x}}_{j}\right\|$ , where $n_{s}$ is the number of samples. Specificity score $\hat{S}\left(n_{m}\right)$ , a measure of the plausibility of SSA model-generated shapes, may be computed as the approximation error (Euclidean distance, in mm) between any randomly sampled shape $y_{A}$ and its nearest training sample $x_{i}$ , summarised as $\hat{S}\left(n_{m}\right)≐\frac{1}{M}\sum_{A=1}^{M}\min_{i}\left\|y_{A}-x_{i}\right\|$ where M is the number of random samples taken. Final parameters were chosen to increase compactness i.e., the morphological variation captured by SSA, as desirable for clustering, despite lowered specificity and generalisation.

### 2.4 Hierarchical clustering

For clarity, clustering analyses are only presented between the original trabeculated LAA surface versus the 4-times reduced dataset. 4-times reduction was chosen as it presents a clear reduction of fine anatomical detail loss, i.e., loss of trabeculae, but largely preserves secondary lobe structure. These two datasets are referred to as the “trabeculated dataset” versus the “simplified dataset” in the results section. Agglomerative hierarchical clustering was applied with MATLAB functions. Complete linkage and correlation distance were chosen; the former to ensure more compact clustering (Ezugwu et al., 2022) and the latter so that anti-correlated objects (i.e., chicken wing-like and non-chicken wing-like shapes) are as far apart as possible (van Dongen and Enright, 2012). The number of PCs accounting for 85% of the total variance (Cangelosi and Goriely, 2007) in the trabeculated dataset was retained for subsequent hierarchical clustering analysis, and the optimal number of clusters was calculated with the silhouette metric (Rousseeuw, 1987), to determine the cut-off value on the dendrograms. Clustering performance evaluation was performed with respect to the previously defined clinical labels, using the adjusted mutual information (AMI) score (Vinh et al., 2010) as the assessment metric. AMI is a measure of similarity (mutual information (MI)) between two labels of the same data, adjusted for chance. For two clusterings U and V:

$$AMI\left(U,V\right)=\frac{MI\left(U,V\right)-E\left(MI\left(U,V\right)\right)}{\text{average}\left(H\left(U\right),\;H\left(V\right)\right)-E\left(MI\left(U,V\right)\right)}$$

## 3 Results

### 3.1 Statistical shape analysis

**FIGURE 5:**
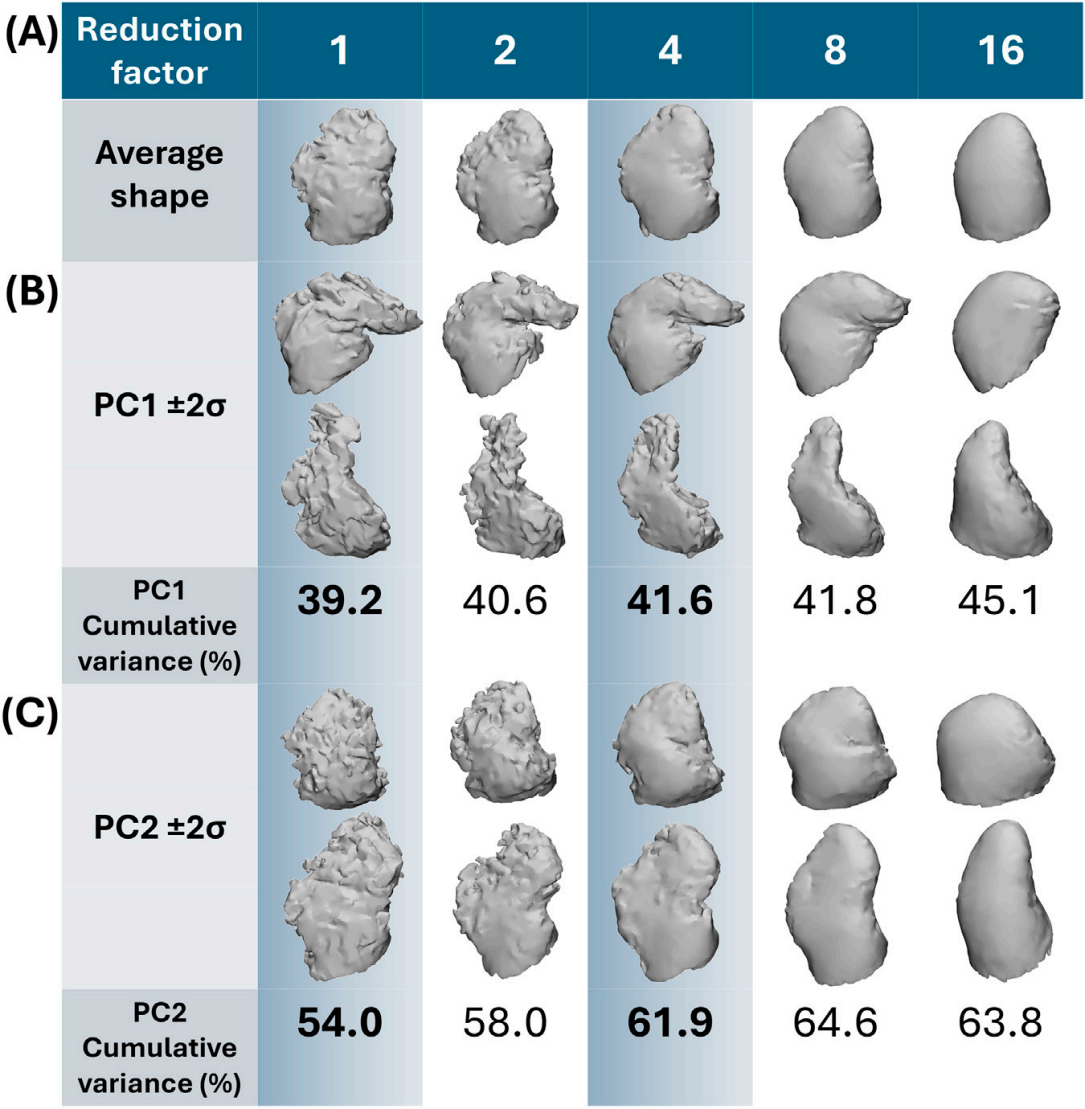
Shape variation captured by the first and second PC. In **(A)** the average shape with increasing reduction factor is presented. In **(B)** moving between 2 standard deviations on PC1 away from the average (±2σ) corresponds to chicken wing-like and non-chicken wing-like shape; with greater cumulative variance captured with increasing reduction with simpler shapes. In **(C)** moving between 2 standard deviations on PC2 away from the average (±2σ) corresponds more to secondary lobe size. Highlighted in blue are the two datasets (fully trabeculated and 4-times reduction) used for clustering comparisons.

**FIGURE 6:**
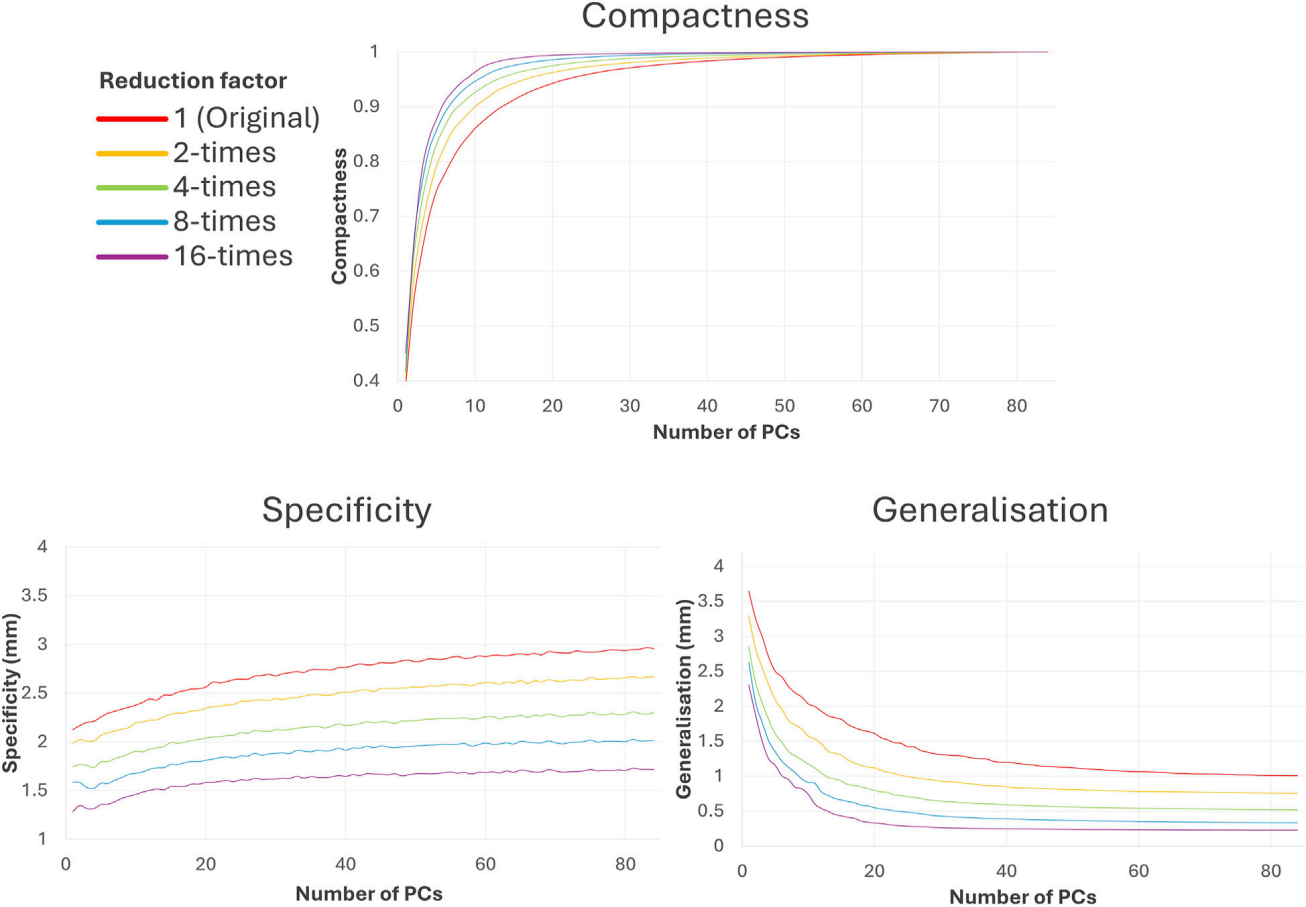
Difference in SSA model evaluation scores compared to the trabeculated dataset with increasing number of PCs. As shown, increasing shape simplification (with increasing reduction factor) increases the amount of morphological variance captured at lower PCs (compactness), decreases the Euclidean distance between a sample shape and its closest training sample (specificity) and improves unseen shape representation (generalisation).

SSA took between 27.8 and 31.3 min to run for each dataset, regardless of anatomical intricacy. The results are presented in terms of visual geometric correspondence (Figure 5) and model evaluation score differences between the trabeculated and simplified datasets with increasing number of PCs (Figure 6).

#### 3.1.1 Geometric correspondence & PCA

For both trabeculated and simplified datasets, most morphological variation (captured by PC1) is between chicken wing-like and non-chicken wing-like shape changes, which matches the observations of previous studies. As presented in Figure 5, moving along the PC1 axis corresponds with shapes more/less similar to the chicken-wing morphology. Moving down PC2 corresponds with smaller/larger secondary lobes. As may be expected, the anatomical detail present in SSA output shapes follows the degree of input shape simplification, with the increase of reduction factor corresponding to a loss in trabecular, surface and lobar definition matching the input datasets. For example, secondary lobes and trabeculae are no longer present by 8-times and 16-times reduction; and even primary lobe morphology is affected.

#### 3.1.2 Shape model evaluation

As may be expected, utilising simpler input shapes translates to easier shape model evaluation. Increasing the reduction factor improves the associated compactness, specificity and generalisation in SSA, as seen in Figure 6. Greater compactness is preserved at lower PCs with increasing reduction factor, which also means that compactness score plateaus earlier. This implies that with simplified datasets, more morphological variation is captured for less PCs. The difference between compactness scores with reduction factor is non-linear; and increasing reduction factor has less effect following 4-times reduction. Specificity error decreases with increasing shape reduction and increases with the number of PCs, implying that more plausible shapes corresponding to each dataset may be generated with more simplified shapes. There is a roughly linear decrease in specificity with increasing reduction factor. Generalisation error (decreasing with the number of PCs) similarly decreases with increasing shape reduction and plateaus earlier, implying that the unseen shapes are better predicted with more simplified datasets. There is a slight non-linear decrease with increasing reduction factor, where greater reduction corresponds with less generalisation decrease.

### 3.2 Hierarchical clusters

**FIGURE 7:**
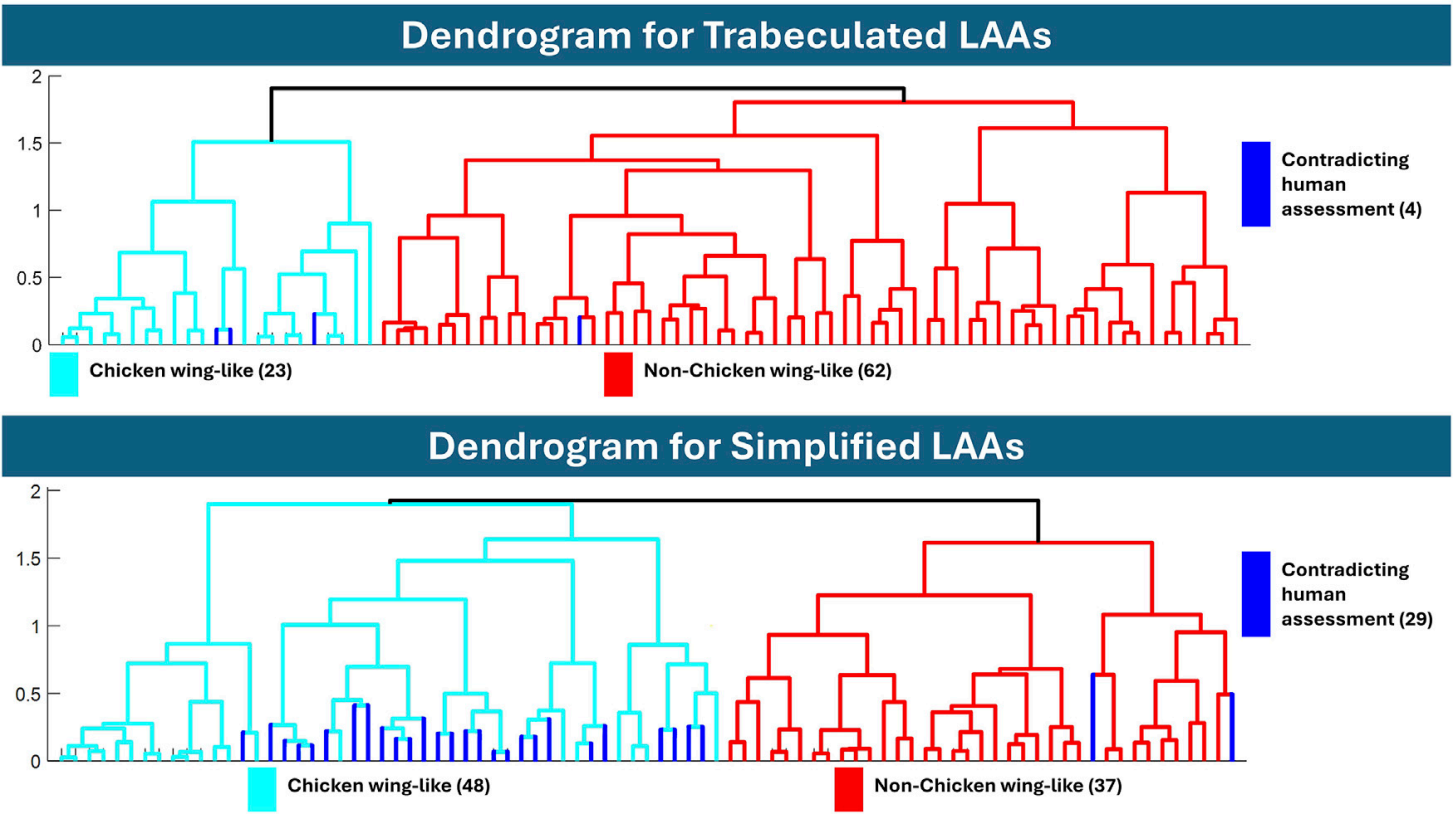
Dendrograms after hierarchical clustering of the trabeculated and simplified datasets. The dendrogram of trabeculated LAA morphologies indicated 23 as chicken wing-like, while the dendrogram of simplified morphology indicated 48. If categorised by a human expert, 21 LAAs are defined as chicken wing, suggesting that the trabeculated dendrogram is closer to human assessment.

**FIGURE 8:**
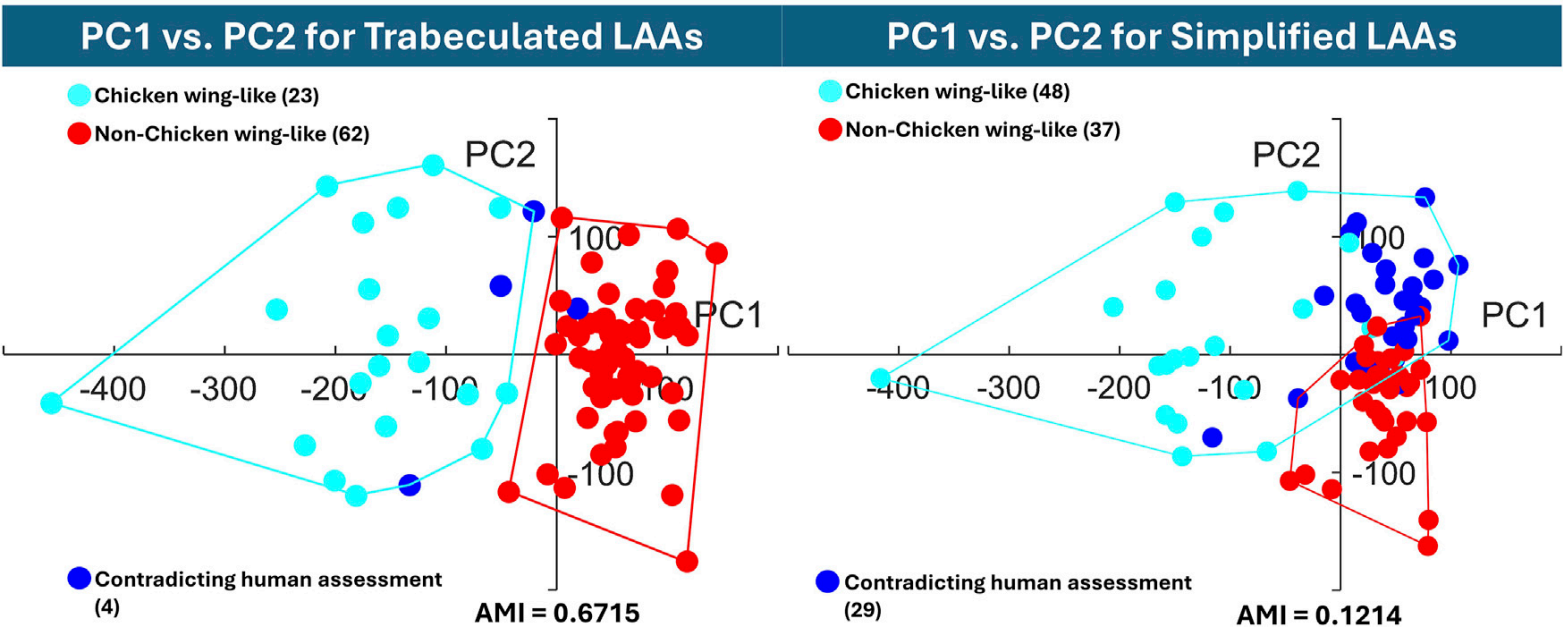
The hierarchical cluster assignments are displayed on the trabeculated PCA distribution (PC1 on the horizontal axis against PC2 on the vertical axis), with AMI according to earlier clinical labels. The graph of the trabeculated dataset shows clear cluster separation between chicken wing (PC1 in the negative direction) and non-chicken wing cases, while the simplified dataset displays high overlap.

Hierarchical clustering results are presented between the original “trabeculated” dataset, and the representative “simplified” dataset of 4-times reduction, with dendrogram results in Figure 7 and visualisation of the data distribution in Figure 8. 10 PCs were found to account for 86.1% of the cumulative variance for the trabeculated dataset, with the optimal number of clusters determined as 2 from a silhouette score of 0.7948. Following the increase in shape model compactness with reduction factor, 10 PCs instead accounted for 92.6% of the cumulative variance for the simplified dataset, with the optimal number of clusters again determined as 2 from a silhouette score of 0.7458. For both datasets, dendrograms with the 2 optimal clusters are presented in Figure 7 and are highlighted on the trabeculated PCA distribution (showing PC1 against PC2) in Figure 8. Figure 8 also records the AMI score of each dataset to the clinical labels.

#### 3.2.1 Dendrogram analysis

Comparing hierarchical clustering of fully trabeculated versus simplified morphologies, the dendrogram for the trabeculated dataset is closer to the current gold standard, i.e., human expert assessment, with 23 LAA morphologies being categorised into a chicken wing-like cluster (with four differences to clinical labels). While computed for 85% cumulative variance, the same clustering is achieved with 90% and 95% cumulative variance. In contrast, 48 LAA morphologies were categorised into the chicken wing-like cluster for the simplified dataset dendrogram (with 29 differences from clinical labels).

#### 3.2.2 Cluster performance evaluation and data distribution

To quantitatively evaluate clustering performance, the AMI score was calculated for both the trabeculated and simplified clusters. With an AMI of 0.6715, the clustering of the trabeculated SSA model PCs is much closer to human assessment than the clustering of simplified SSA model PCs with a score of 0.1214. To visually present the clustering performance, the obtained hierarchical clusters are highlighted on their original PCA distributions for two axes (PC1 against PC2) in Figure 8. As shown, there is clearer cluster separation for the trabeculated dataset, where the chicken wing-like cluster is more dispersed than the non-chicken wing-like cluster. In contrast, the simplified dataset presents a strong overlap relative to human assessment. This overlap is mainly in the positive PC1 and PC2 directions, corresponding to non-chicken wing-like shapes and to smaller secondary lobes respectively, as presented in Figure 5C.

## 4 Discussion

### 4.1 Principal findings

With selected parameters for SSA and clustering, results suggest that LAA shape categorisation via hierarchical clustering performs better with preservation of full anatomical details (the “trabeculated dataset”) than with trabecular detail loss (called the “simplified dataset”). While greater LAA anatomical simplification directly corresponds with better SSA model evaluation scores for compactness, specificity and generalisation (Figure 6), it was hypothesised that the loss of trabecular detail affects the preservation of morphological variation pertinent for LAA shape categorisation (Figures 3 and 5).

Between the trabeculated and simplified datasets, the improvement to SSA evaluation with reduction at the 10 PCs used for subsequent clustering is as follows: +0.065 compactness, −0.47 mm specificity and −0.86 mm generalisation (Figure 6). This is expected as the shape simplification process has led to a decrease in anatomical trabeculae and lobar definition that would have accounted for greater morphological difference between shapes. This implies that increasing anatomical simplification increases both the SSA model’s ability to plausibly generate LAA shapes within simplified datasets and how well the model may generally represent unseen LAA shapes. However, as greater reduction by 8-times and 16-times visually affects even LAA lobar structure (Figure 3), it is thought that the greater anatomical simplification affects the geometric correspondence between shapes (Figure 5). Therefore, reduction by 4-times was selected as the simplified dataset for subsequent clustering comparisons. For visual comparison between PC1 and PC2 for these two datasets (Figure 5), PC1 captures chicken wing-like and non-chicken wing-like bending angle. PC2 instead describes LAA shapes with smaller or larger secondary lobes.

In contrast, increasing LAA reduction in SSA lowered clustering performance. The simplified model clusters, with a low AMI score of 0.1214, are mainly overlapping in the +PC1 and +PC2 quadrant (Figure 8), with 29 shapes being assigned differently to human assessment. This suggests that while + PC2 is associated with smaller secondary lobes, the inclusion of secondary lobe detail, e.g., trabeculae, better separates chicken wing-like shapes. On the trabeculated model clusters of Figure 8, the higher AMI score of 0.6715 corresponds with good cluster separation on the trabeculated PCA distribution, with only four shapes assigned differently to human assessment. This clustering is also more stable, with the same clusters being achieved for 90% and 95% cumulative variance. Therefore, these results may justify the preservation of intricate anatomical details, particularly LAA trabeculae, for shape categorisation with hierarchical clustering, despite improvements to pure SSA evaluation scores. In terms of computation time, SSA was less affected by the anatomical differences between datasets rather than the parameters chosen, taking between 27.8 and 31.3 min to run on the same AMD Ryzen 9 7950X3D 16-Core Processor, 4201 Mhz, 16 Core(s), 32 Logical Processor(s).

### 4.2 Broader research context

#### 4.2.1 Clinical LAA shape categorisation schemes

Despite its popularity, conventional LAA classification (into four shape classes, chicken wing, cactus, cauliflower and windsock) is highly subjective, with a clinical study suggesting full shape category agreement between three observers was only reached in 28.9% of 2,264 cases (Wu et al., 2019). Other studies suggest the presence of 2–8 LAA classes depending on additional study aims. Some studies with only 2 shape classes separate LAAs into lower versus greater risk, based on the number of lobes (He et al., 2020) or with/without chicken wing-like bending (Yaghi et al., 2020). A clinical study suggests that LAA morphologies are instead combinations of up to 8 qualitative lobe shapes, preferring visual lobe classification instead of general shape categorisation (Beutler et al., 2014). With special focus on quantitative anatomical measurements not just of the LAA but of adjacent structures and the body, LAA clinical classification may even extend to 7 shape categories with 6 subtypes (Li et al., 2015). These studies highlight the sheer diversity of LAA shape complexity even without consideration of finer anatomical details, and the need for an objective shape categorisation from clustering analysis of SSA models, as employed here. As our study currently focusses on chicken wing-like and non-chicken wing-like shape categorisation, this is more similar to the simplified clinical categorisation with/without chicken wing-like bending (Yaghi et al., 2020), but without needing human intervention.

#### 4.2.2 Applications of anatomical detail in LAA meshes

While clinical categorisation schemes are useful for simplified understandings of the connection between LAA morphology and thrombosis risk, the subjectivity of such classifications (Wu et al., 2019) may subsequently lead to inaccurate risk stratification. Furthermore, clinical categorisation typically does not consider the impact of intricate anatomical details, which may be difficult to measure manually.

A more in-depth comprehension of the LAA shape-haemodynamic relationship requires 3D LAA meshes, which provide 3D anatomical variation that is useful for computational modelling. While many studies do not consider intricate anatomical details, studies that do consider such impact (Musotto et al., 2022) suggest that trabeculae play an important role in LAA haemodynamics, by reducing LAA blood washout.

#### 4.2.3 Other LAA SSA studies

Previous LAA SSA studies aim to objectively define LAA shape categories beyond current clinical capabilities, although no study to date is built from LAA morphology with full anatomical detail preservation. Explicit LAA SSA is typically based on the point distribution model (PDM) (Cootes et al., 1995), where correspondence between shapes is defined by the automatic placement of points across surfaces. The most studied optimisation scheme for LAA explicit correspondence is the entropy scheme used in ShapeWorks (Cates et al., 2017) (applied on both the LAA only (Goparaju et al., 2022; Goparaju et al., 2018) and for the conjoint left atria with LAA (Bieging et al., 2021; Adams et al., 2023; Adams et al., 2022; Cates et al., 2015)), where increasing particle correspondence may be iteratively initialised and optimised with regularisation parameters. Alternatively, explicit LAA SSA studies may determine initial point correspondence through Markov Random Field regularisation (Juhl et al., 2024; Slipsager et al., 2019) of the correspondence vector fields between source and target shapes (Paulsen et al., 2003). LAA SSA may also be applied implicitly on both the LAA only (Goparaju et al., 2022; Ahmad et al., 2024; Goparaju et al., 2018) and for the conjoint left atria with LAA (Corrado et al., 2020). Implicit approaches typically rely on the optimisation of deformations in a Riemannian space to warp shapes into others (Bône et al., 2018; Hartman et al., 2023). Established frameworks, such as Deformetrica (Bône et al., 2018), have been used (Goparaju et al., 2022; Goparaju et al., 2018), and recent works have also experimented with dedicated frameworks (Hartman et al., 2023) applied specifically to the LAA (Ahmad et al., 2024). However, to our knowledge, such methods do not allow the high complexity of the LAA surfaces to be considered. Of all the studies mentioned, the most recent advances in LAA SSA (Juhl et al., 2024; Ahmad et al., 2024) have focused mainly on chicken wing and non-chicken wing shape classification, proposing that more in-depth shape categorisation may fit within this overarching division.

Lower LAA morphological complexity may be a consequence of lower image input resolution (Cates et al., 2015), or that images have been intentionally “downsampled” to reduce noise (Juhl et al., 2024) e.g., for deep learning segmentation (Juhl et al., 2024; Ahmad et al., 2024). As discussed earlier, inclusion of fine LAA morphological detail not only improves thrombosis risk assessment of AF patients (Musotto et al., 2022) (the primary reason for LAA shape analysis) but may also be discriminatory for shape categorisation (Martorana et al.). Therefore, previous SSA studies may be limited in clinical applicability.

#### 4.2.4 Computational categorisation methods in LAA SSA

Current shape categorisation methods in LAA SSA may utilise hard and soft clustering approaches, as well as non-clustering dimensionality reduction. Hard clustering on LAA SSA has been approached with k-means (Goparaju et al., 2018) and hierarchical clustering with additional multidimensional scaling (Ahmad et al., 2024), in comparison to our study focussing on hierarchical clustering only. A hard clustering approach may be more useful for the analysis discussed in this study, where categorisation between chicken wings and non-chicken wings should present less overlap. Soft clustering of LAA SSA, where overlap may be considered, has been approached with Gaussian Mixture Modelling (Juhl et al., 2024; Slipsager et al., 2019). Alternatively, another study suggests the use of t-stochastic Nearest Neighbour Embedding for their LAA SSA (Goparaju et al., 2022), which may be useful to display trends not visible with clustering methodologies.

To our knowledge, no other LAA SSA studies have presented the numerical efficacy of their shape categorisation with respect to human evaluation, so this is difficult to compare to other studies. In this work, AMI was chosen to evaluate cluster performance over rand-index scoring as unequal cluster sizes were expected (van der Hoef and Warrens, 2019), with only 21 of the 85 segmented LAAs having been expertly classified as chicken wing morphology earlier. Furthermore, as an adjustment of the regular mutual information metric, chance clustering assignments are accounted for.

### 4.3 Strengths and limitations of study

The applicability of the proposed LAA SSA model and clustering is limited by the analysed number of anatomies in the original dataset. This is particularly important for highly diverse anatomies such as the LAA, where it is highly likely for morphologies to demonstrate categorical variance beyond subjective clinical classification, even without considering fine anatomical details. In comparison with other LAA works, the number of LAAs utilised in our study (85 in total) lies between other studies, which can vary from 20 (Ahmad et al., 2024) to 130 (Goparaju et al., 2022). However, no other SSA study to our knowledge has preserved our level of LAA anatomical detail, which is the basis for this study.

Some limitations are related to operator-dependent steps in our workflow. Firstly, the manual left atrial segmentation (prior to fully automatic LAA detection) requires user definition of contrast threshold (aided by the additional mathematical CNR measurement protocol) and human effort and time to ensure segmentation is not affected by unwanted imaging artefacts. The second operator-dependent step is the clinical classification used to obtain the clinical labels to which clustering is compared in AMI scoring; clinical subjectivity was minimised in this study by focusing clinical labels to chicken wing versus non-chicken which is known to present the greatest morphological difference of bending angle (Yaghi et al., 2020). Two of the aforementioned LAA SSA studies have aimed to tackle the segmentation problem via deep learning (Juhl et al., 2024; Ahmad et al., 2024); however, as already stated, these works do not fully capture the same level of anatomical detail, presenting very smooth meshes, i.e., without trabeculae. Furthermore, the fully automatic LAA detection of the ostial plane utilised in our study may be further advantageous over both these studies that either cut the shape where it is narrowest (Juhl et al., 2024) (which describes an anatomical region generally different from the ostium definition) or perform manual clipping of left atrial meshes (Ahmad et al., 2024).

Finally, it should be noted that while a pixel spacing of 0.488 mm from CT is high for conventional clinical scans, even higher resolutions exist for alternative *ex-vivo* imaging-based studies e.g., microCT, synchrotron-based or photon-counting CT imaging. This study indicates that clustering of anatomies acquired with smaller pixel spacing performs significantly better than lower resolutions, which suggests that even higher resolution scan data could improve the results further. To increase the reliability and statistical significance of this work, it would be beneficial to incorporate more LAA morphologies in the SSA performed; however, it was not possible to include datasets acquired from publicly accessible databases (Atria Segmentation Challenge 2018; Karim et al., 2018) as they either did not match the imaging modality and/or the required resolution.

## 5 Conclusion and future works

SSA studies for clustering analysis of highly diverse anatomies, particularly the human LAA, may suffer from analytical disparities and therefore clinical relevance due to major differences in anatomical detail preservation. Following robust image and mesh processing, this study applies SSA and clustering analysis to 5 LAA datasets (each composed of 85 shapes), sequentially reduced in anatomical detail. While evaluation scores of SSA metrics of compactness, specificity and generalisation suggest lower resolutions may improve LAA shape representation of such simplified anatomies, it should also be recognised this better representation may not correlate with improved LAA shape categorisation. The cluster performance scores suggests that clustering for LAA shape categorisation benefits from greater preservation of anatomical detail (beyond the level conventionally preserved in LAA SSA). Future work could improve upon binary categorisation (i.e., chicken wing-like vs. non-chicken wing-like) by adjusting the dendrogram cut-off thus leading to smaller morphological sub-groups. In preserving trabeculae, this study advances towards connecting SSA anatomical detail to thrombosis risk categorisation.

## Data Availability

The datasets presented in this article are not readily available because of concerns regarding participant/patient anonymity. Requests to access the datasets should be directed to the corresponding author.
